# Epidemiology of Angina Bullosa Hemorrhagica: A Retrospective study

**DOI:** 10.22038/ijorl.2024.71853.3442

**Published:** 2025

**Authors:** Meenakshi Sachdeva, Pratik Kumar, Keshav Gupta

**Affiliations:** 1 *Department of Dermatology, Deep Chand Bandhu Hospital, Ashok Vihar, New Delhi, India. *; 2 *Department of Otorhinolaryngology, Maulana Azad Medical College, New Delhi, India.*; 3 *Department of Otorhinolaryngology, Saraswathi Institute of Medical Sciences, Hapur, U.P., India.*

**Keywords:** Angina bullosa hemorrhagica, Local trauma, Oral blisters, Diabetes

## Abstract

**Introduction:**

Angina Bullosa Hemorrhagica (ABH) is a rare condition characterized by hemorrhagic blisters and is often asymptomatic. These lesions appear more commonly in the oral cavity and oropharynx and are often misdiagnosed. A retrospective cross-sectional study was performed in clinically confirmed cases of ABH to study its epidemiology, etiology and presentation in a tertiary care hospital in Southern Asia.

**Materials and Methods:**

Total of 8 patients of ABH were evaluated and included in the present study. All clinical data and track records were assessed by the medical records department. Cases were studied and results were interpreted. Results: Total of 8 cases were enrolled with male-to-female ratio of 0.6:1 and middle age as the most common age of presentation. Buccal mucosa was the most common site involved with average lesion size of 1.6 cm. Masticating trauma was the most common etiological risk factor. Majority patients were asymptomatic with spontaneous resolution of lesions in all the cases.

**Conclusion:**

Due to smaller number of studies reported, the lesions of ABH remained poorly understood with uncertain etiology. The knowledge of characteristic clinical features of the lesion and pattern of spontaneous healing is of utmost importance as a lesion of ABH can share some features with other serious disorders, thereby delaying the diagnosis. A thorough clinical history and examination of the lesion should always be done to establish an accurate diagnosis. Due to its completely benign nature, proper counseling of the patients must be ensured for better patient compliance.

## Introduction

Angina Bullosa Hemorrhagica (ABH) is an extremely rare benign lesion that is poorly described in the literature with uncertain etiology & a prevalence rate of 0.03% (1,2). The term “Angina Bullosa Hemorrhagica” was first described by Badham in the year 1967 (3). He described the lesion as an acute, blood-filled vesicle more commonly seen in oral cavity and oropharynx. These lesions are painless and are mostly asymptomatic. Similar lesions were also described by Balina, earlier in 1933 under the term Traumatic Oral Hemophlyctenosis (TOH) (4). ABH is more commonly seen in middle-aged population. Although the etiology is unknown, these lesions are thought to precipitate after masticating trauma. Other factors like consuming hot drinks and oily food items can also contribute to the formation of ABH. This is one of the reasons that ABH is more commonly seen in the Indian subcontinent. Although ABH is a benign clinical entity, it remains underdiagnosed in many cases leading to treatment delay. Close observation and follow-up are necessary for complete cure of the lesion. In this article, a cross-sectional study was performed on 8 patients presented with ABH highlighting their epidemiology, risk factors and characteristics of the lesion. 

***Objectives***

A retrospective cross-sectional study was performed in clinically confirmed cases of ABH to study its epidemiology, etiology, presentation and risk factors associated with the disease in a tertiary care hospital in Southern Asia.

## Materials and Methods

A retrospective cross-sectional study was done in a tertiary care hospital in southern Asia with total of 5000 cases who presented with oral lesions between 2019-2023. Inclusion criteria for ABH were according to the Ordioni et. al. with clinically notable hemorrhagic lesion, oral or oropharyngeal location, presence of triggering factor or food intake, recurrence, favorable resolution without scar, minimal or no pain on presentation and normal coagulation profile. (5) All the confirmed cases diagnosed as ABH were distinguished and evaluated. Data collection and analysis were done retrospectively by tracking the clinical records from medical record department, available documentations from the patients and questionnaires & surveys from the patients. Further information was collected from the oral histories provided by the patients. A total of 8 patients of ABH fulfilling the Ordioni et al criteria were included in the study. The clinical symptoms of the patients were assessed using the questionnaire containing the common symptoms like pain, burning sensation or difficulty swallowing. 

## Results

Out of total 5000 patients studied retrospectively in a cross-sectional study at a tertiary care hospital, 8 patients of ABH were diagnosed and included in the study (Table 1).

**Table 1:** Clinical details of patients suffering from Angina Bullosa Hemorrhagica (ABH)

**Case**	**Age** **(years)**	**Gender**	**Location**	**Size** **(cm)**	**Aetiology (probable)**	**Symptoms**	**Resolution**	**Underlying Disease**
1	45	M	Right buccal mucosa	2	Dental trauma	asymptomatic	Spontaneous (7 days)	none
2	35	F	Ventral aspect tongue	1	hot drinks	asymptomatic	Spontaneous (15 days)	none
3	55	F	hard palate	2.5	Steroid inhalers	asymptomatic	Spontaneous (14 days)	Asthma, DM
4	47	M	Floor of mouth	1	Constipation and Gastric Reflux, Oil and spices	asymptomatic	Spontaneous (11 days)	none
5	31	F	Left buccal mucosa	2	Dental trauma, hard food items	Mild pain	Spontaneous (9 days)	none
6	43	M	Floor of mouth	2	Dental trauma	asymptomatic	Spontaneous (8 days)	none
7	49	F	Soft palate	1	Hard food items	asymptomatic	Spontaneous (20 days)	none
8	55	F	Right buccal mucosa	2	Dental trauma, hot food items	Mild pain	Spontaneous (9 days)	DM, HTN

Out of a total 8 patients, 3 were males and 5 were females. The mean age of presentation was 45 years. The study concluded that females were more commonly involved as compared to males with male-to-female ratio of 0.6:1. The mean age of presentation of ABH in this study concludes that ABH is more commonly seen in middle-aged population. The most common site of lesion was the involvement of buccal mucosa. Other sites of involvement were the soft palate, hard palate, ventral aspect of tongue and floor of mouth. Majority of lesions of ABH corresponds to approx. 2 cm in size. The average size of lesion in this study was 1.6 cm. The present study concludes that the majority of factors can play a role in the etiology of ABH as masticating trauma, consuming hot and oily food items, consuming rough or hard food items, steroid inhalers or gastro esophageal reflux disease (GERD). The most common and probable etiological factor was dental trauma due to injury of oral cavity mucosa by tooth during mastication. This can be justified by the fact that during mastication, the buccal mucosa is more likely to get injured which accounts for the most common site for the development of ABH. Majority of patients with ABH reported no symptoms on presentation. Two out of 8 patients reported only mild pain during mastication as a symptom. The study showed that almost all of the lesions of ABH regressed spontaneously without need for any medication. The variation in the duration of spontaneous healing of the lesion was observed with a minimum duration of 7 days to a maximum of 20 days. The average time of spontaneous healing of lesion of ABH without any medication was observed up to 12 days. The minimum time for spontaneous healing was observed for lesions involving the buccal mucosa (7 days) and maximum time was observed for lesions involving soft palate (20 days). Majority of patients with ABH had no underlying disease except 2 out of 8 had diabetes. Out of these 2 patients, one had asthma and other had hypertension. Both patients were on oral hypoglycemic medications with controlled blood sugar levels. 

## Discussion

**Fig. 1:**
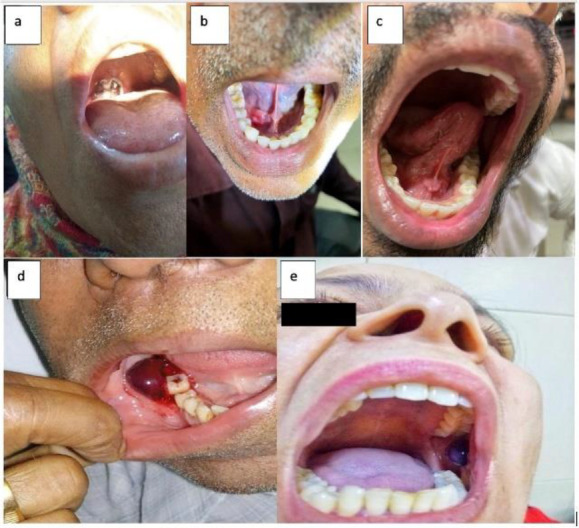
(a) Painless ulcer in early stage of resolution of ABH; (b) & (c) Spontaneous regression of lesion during later stage; (d) Reddish blood-filled blister in acute stage; (e) Bluish red blister with smooth and regular surface in acute stage.

The term angina bullosa hemorrhagica (ABH) was first coined by Badham in 1967 (3). It is an acute onset, blood-filled blister that occurs in the oral cavity and oropharynx. These lesions occur spontaneously and regress on their own, most of the time without leaving any scar. These lesions can occur at different locations with variable presentation (Figure 1). 

ABH most commonly occurs over buccal mucosa and soft palate followed by involvement of the tongue, gingiva-buccal sulcus and lips (6). Our study showed the buccal mucosa as the most common site of involvement of ABH. Isolated involvement of hard palate, gums or lips is seen less frequently. ABH lesions appear as blood-filled bullae which can be solitary or multiple, recurrent without any signs of inflammation. ABH is more commonly seems to involve middle-aged and elderly population. Our study also had middle age as the most common presenting age with similar results comparable to another study with mean age of 54.8 years (7). Our study had the male-to-female involvement of 0.6:1. ABH blister can vary from small to large size lesions thereby producing variety of symptoms like pain, difficulty in chewing or burning sensation of the involved mucosa. Large-sized ABH bullae in the oropharynx can even produce the feeling of suffocation in patients (3). Our study had the average size of the lesion corresponding to 1.6 cm. A similar study in the past had the lesion size ranging from 1.0 to 3.5 cm, with a mean of 2.0 cm (8). The etiology of ABH is unclear, however, many risk factors are thought to trigger this condition. Trauma of the oral cavity mucosa by tooth during mastication is the most common etiological factor. This can be explained by the fact that during mastication, there is an increase in the blood flow to the mucosa of the oral cavity and oropharynx due to the parasympathetic reflex vasodilation (8). Any minor injury or trauma to the oral cavity or oropharyngeal mucosa can lead to activation of the clotting factors and organization of the scar into a blood-filled blister. Other risk factors include excessive consumption of hard food items, hot drinks or spicy food that can precipitate the ABH. One possible explanation can be due to minor mucosal damage of the oropharyngeal mucosa by hard or hot food items. Dental procedures also increase the risk for ABH (9). A case of a post-operative ABH has also been reported to be caused by intubation and extubation (10). 

The possible explanation for this can be due to the minor mucosal injury that can make oral mucosa more susceptible to developing acute onset, blood-filled blister. Our study showed that the majority of patients had no underlying disease except for two patients showing the history of diabetes. The blood sugar levels of these two patients were, however, under well controlled limits. This shows that well-controlled diabetes as an underlying disease may not play a significant role in the formation of ABH as the other 6 patients had no history of diabetes. However, some studies suggest hypertension as a systemic illness may precipitate ABH (8). Our case did not have any significant history of hypertension as the underlying cause except for a single case with a well-controlled blood pressure on medications. Our study concludes that hypertension under control may not play any significant role in the progression of the lesion of ABH. Excessive use of Steroid inhalers by asthmatic patients is also thought to precipitate these oral lesions (11).

**Fig. 2:**
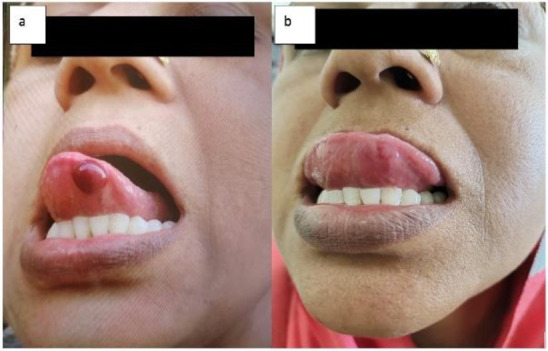
(a) ABH lesion over ventral aspect tongue; (b) spontaneous resolution of lesion without leaving any scar.

Excessive and chronic use of steroids alters the collagen formation which subsequently results in mucosal atrophy that increases the risk for formation of blood-filled bullae in the oral cavity and oropharynx (12). The diagnosis of ABH can be made clinically due to the pattern of the lesions. The blisters of ABH appear more commonly over buccal mucosa and soft palate as smooth, reddish to bluish, blood-filled vesicles, which are often painless, acute in onset, recurrent in nature, non-fluctuant and non-compressible. These lesions heal spontaneously within 1-2 weeks leaving a painless ulcer during an early stage of resolution. The lesion gradually regresses in size in the later stage of resolution. A similar pattern of resolution was noted in other studies also (13). The duration for complete resolution of the lesion is variable and usually heals without leaving any scar (Figure 2).

Patients with ABH have normal hematological parameters. Histopathological examination (HPE) of the lesion further aids in the confirmation of the diagnosis to rule out other blistering conditions of oral cavity like amyloidosis, cicatricial pemphigoid or epidermolysis bullosa acquisita. Histopathological features of ABH include parakeratotic epithelium with a subepithelial separation from the underlying lamina propria. Superficially located vesicles filled with erythrocytes and fibrin are seen. The inflammatory cell infiltrate, when present consists primarily of lymphocytes (13). 

Conservative management is the preferred treatment modality for small to moderate size blisters of ABH of the oral cavity and oropharynx (6-8). Thorough clinical history and examination of the lesion favor the diagnosis, which can be supplemented with a biopsy of the lesion for confirmation. In our study also, all the lesions were histopathologically confirmed to establish the accurate diagnosis and to rule out other similar lesions of oral cavity and oropharynx. Complete blood tests including coagulation profile and viral markers should always be done to rule out any bleeding disorders. Other dermatological lesions if suspected, should always be ruled out. A large blister of ABH at the level of oropharynx can be drained surgically if choking episodes are present. In our study, all the lesions healed spontaneously without any medication with variable time intervals that was consistent with the similar results from the previous studies in the past (10-12). 

Due to its benign nature and spontaneous resolution of the lesion, most patients generally fail to reach to the healthcare providers which can be one of the factors for underdiagnosis or delay in the treatment. Patients should always be counseled for the benign nature of the disease. Although the lesion is mostly asymptomatic and regresses spontaneously, patients who present with mild symptoms can be treated with chlorhexidine mouthwash and maintaining oral hygiene that can speed up the recovery of the lesion. 

## Conclusion

Due to a smaller number of studies reported, the lesions of ABH remained poorly understood. The knowledge of characteristic clinical features of the lesion and the pattern of spontaneous healing is of utmost importance as the lesions of ABH can share some features with other serious disorders, thereby delaying the diagnosis. This entity is more commonly seen in middle-aged with female preponderance and buccal mucosa as the most common site. 

A thorough clinical history and examination of the lesion should always be done to establish an accurate diagnosis. Almost all the lesions heal spontaneously leaving no scar, making it an underdiagnosed entity. Due to its completely benign nature, proper counseling of the patients must be ensured for better patient compliance. 
